# mRNA Metabolism and Hypertension

**DOI:** 10.3390/biomedicines11010118

**Published:** 2023-01-03

**Authors:** Martina Zappa, Paolo Verdecchia, Antonio Spanevello, Michele Golino, Fabio Angeli

**Affiliations:** 1 Department of Medicine and Surgery, University of Insubria, 21100 Varese, Italy; 2 Fondazione Umbra Cuore e Ipertensione-ONLUS, 06100 Perugia, Italy; 3 Division of Cardiology, Hospital S. Maria della Misericordia, 06100 Perugia, Italy; 4 Department of Medicine and Cardiopulmonary Rehabilitation, Istituti Clinici Scientifici Maugeri IRCCS, 21049 Tradate, Italy

**Keywords:** hypertension, blood pressure, mRNA, metabolism, chronic disease

## Abstract

Hypertension is the most frequent cardiovascular risk factor all over the world. It remains a leading contributor to the risk of cardiovascular events and death. In the year 2015, about 1.5 billion of adult people worldwide had hypertension (as defined by office systolic blood pressure ≥ 140 mmHg or office diastolic blood pressure ≥ 90 mmHg). Moreover, the number of hypertensive patients with age ranging from 30 to 79 years doubled in the last 30 years (from 317 million men and 331 million women in the year 1990 to 652 million men and 626 million women in 2019) despite stable age-standardized prevalence worldwide. Despite such impressive growth, the proportion of controlled hypertension is very low. A better understanding of the pathogenesis of hypertension may contribute to the development of innovative therapeutic strategies. In this context, alterations of the messenger RNA metabolism have been recently evaluated as contributors to the pathogenesis of hypertension, and pharmacological modulation of RNA metabolism is under investigation as potential and novel therapeutic armamentarium in hypertension.

## 1. Introduction

Hypertension is the most frequent non-communicable and chronic disease all over the world and it remains a leading risk factor for cardiovascular events and death [1,2,3,4,5]. A study including 1479 population-based surveys (for a total of 19.1 million adult participants for whom blood pressure [BP] was measured) estimated the prevalence of hypertension at 1.13 billion in 2015 globally (with a prevalence of more than 150 million in Central and Eastern Europe) [6].

Approximately 7.6–9 million premature deaths per year are attributed to uncontrolled hypertension [7,8] and a systolic BP (SBP) ≥ 140 mmHg explains about 70% of the burden of mortality and morbidity worldwide [9,10,11,12].

Despite the epidemiological impact of hypertension, national cross-sectional surveys, community studies, epidemiological investigations, health maintenance organizations, and reports of physician office practices showed that the proportion of well-controlled hypertension remains very low worldwide [13]. Specifically, it has been estimated that the proportion of uncontrolled hypertension approaches 23% and 18% in women and men, respectively [6].

Furthermore, the pathogenesis of primary hypertension is still poorly understood and the last few years are characterized by an impressive paucity of new and innovative clinical studies in the treatment of hypertension [14].

In this context, it is now well recognized that hypertension is the result of numerous genetic factors that have multiple compounding effects on the cardiovascular structure and function. Moreover, alterations in the metabolism of ribonucleic acid (RNA) have been recently invoked as a potential key factor in the etiology of hypertension.

The main aim of this review is to summarize accrued data on the role of alterations of messenger RNA (mRNA) metabolism in the pathogenesis of hypertension. We also discuss the potential implications of the pharmacological modulation of mRNA metabolism in hypertension.

## 2. Pathophysiology of Hypertension

BP is dependent on the balance between peripheral vascular resistance and cardiac output. The physiologic mechanisms that ensure the maintenance of BP values and pressure variability are numerous: the arterial baroreceptor system, the sympathetic nervous system (SNS), the renin-angiotensin-aldosterone system (RAAS), endothelial vasodilation and vasoconstriction factors, and the role of the kidneys in responding with appropriate natriuresis to varying BP levels.

Many factors may contribute to the pathogenesis of hypertension. Genetic factors, activation of neuro-hormonal systems, obesity, and environmental factors such as stress and increased salt in the diet play a key role [15,16].

**Figure 1:**
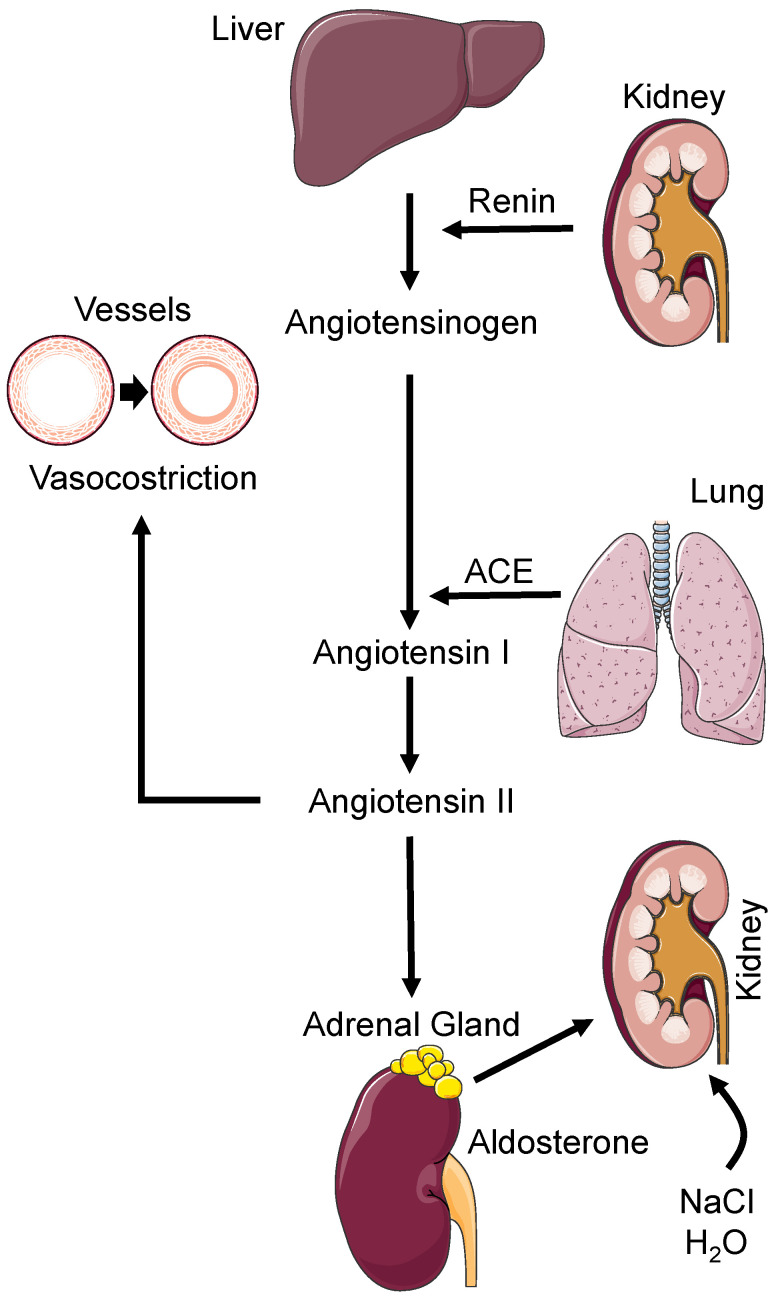
Schematic representation of the renin-angiotensin-aldosterone system. ACE: Angiotensin Converting Enzyme, H_2_O: chemical formula for water, NaCl: chemical formula for sodium chloride. See text for details.

Among the biological mechanisms, the activation of the RAAS exerts a pivotal role (Figure 1).

RAAS promotes the maintenance of BP values through the cascade enzyme that leads to the production of angiotensin II [17,18]. The effects of angiotensin II are mediated mainly by two classes of receptors, AT1 (angiotensin II type 1 receptor) and AT2 (angiotensin II type 2 receptor). Most of the effects of angiotensin II appear to be mediated by AT1 receptors expressed in all tissues implicated in the pathogenesis of hypertension, such as the circulatory system, the nervous system, and the kidneys. Activation of these receptors causes increased inflammation, oxidant production, and cell proliferation resulting in vascular remodeling. The activation of AT2 receptors, expressed at low levels in renal, cardiac, and mesenteric vessels, results in the opposite effects, such as inhibition of cell growth, apoptosis, and vasodilation. Angiotensin II is able to achieve feed-back with the nervous system by acting on the vasomotor nuclei and promoting stimulation of the sympathetic system [17,18].

Ultimately, the RAAS system is closely involved in the pathogenesis of hypertension through various mechanisms ranging from vasoconstriction, vascular and cardiac remodeling, stimulation of renal sodium reabsorption, and activation of the SNS [19]. Inhibition of this system is currently one of the therapeutic strategies for the prevention of organ damage and for the reduction of BP values [14,20,21,22,23,24,25,26,27].

## 3. Genetics of Hypertension

The genetic determination of hypertension in humans has proven challenging. Evangelou and coworkers reported the largest genetic association study of BP traits (i.e., SBP, DBP, and pulse pressure [PP]) to date, including over 1 million subjects of European ancestry [28]. Of note, the identification of 535 novel BP loci offers new specific biological insights into BP regulation, highlighting shared genetic architecture between BP regulation and lifestyle exposures [28].

Recent technological developments have given rise to what are now called omics sciences, a group of disciplines including epigenomics, genomics, transcriptomics, proteomics, and metabolomics. Omics approaches offer the advantage of enabling the identification of new mechanisms and pathways that allow for further characterization of the pathophysiology of the disorder. Moreover, the integration of these mechanisms helps with a comprehensive assessment of the processes by which deoxyribonucleic acid (DNA) is transcribed into ribonucleic acid (RNA), which is translated into proteins that regulate downstream metabolism.

With the development of new tools for genetic studies, major efforts are being directed to investigate the potential genetic causes of primary hypertension. In particular, technology in genome sequencing enabled the growth of specific studies in this area.

Several gene markers and loci associated with hypertension susceptibility have been identified. A great effort was made to identify genetic variants affecting SBP and DBP through Genome-Wide Association Studies (GWAS), a valuable approach to type hundreds of thousands of Single Nucleotide Polymorphisms (SNPs) [29,30]. Despite this, GWAS in which hundreds of thousands of common genetic variants are genotyped and analyzed for BP association have shown limited success in the identification of genes contributing to hypertension [29,30].

Each gene variant has a relatively weak impact on BP levels; however, it has a potential significant effect on the rise in BP when several variants act together in the presence of the necessary environmental conditions. Thanks to the establishment of consortia in recent years, many studies have been published, leading to the identification of more than 100 SNPs implicated in BP levels [29,31,32,33]. In particular, Hoffmann and coworkers identified 39 new loci among 75 genome-wide significant loci (*p* ≤ 5 × 10^−8^) and they found that about 26% of the SNPs had significantly different normalized effect sizes for SBP and DBP (*p* < 0.00013); for 57.4% of these SNPs, the normalized effect was greater for SBP than for DBP [33].

As reviewed in 2007 by Seidel and Scholl [34], for hypertension, there were no SNPs with a significance below 5 × 10^−7^; however, the number and distribution of association signals in the range from 10^−4^ to 10^−7^ was quite similar to that of the other diseases, including type 1 and type 2 diabetes, coronary artery disease, Crohn’s disease, and rheumatoid arthritis [34].

## 4. The Role of RNA

RNA is a nucleic acid that is present in the majority of living cells and it is composed of nucleotides (ribose sugars attached to nitrogenous bases and phosphate groups). RNA mostly exists in the single-stranded form; however, there are special RNA viruses with double-stranded RNA. RNA synthesis is usually catalyzed by an enzyme, RNA-polymerase, using DNA as a template, a process known as transcription.

The central dogma of molecular biology illustrates the importance of mRNAs as the central point for the passage of genetic information to the proteome/metabolome world that determine the various functional outcomes at the cellular and organ level. In perfect harmony with the transcription process operated by the RNA polymerase II complex, mRNA transcripts are immediately modified through different processes including classical 5′-capping, intron splicing, and 3′-polyadenylation to produce mature mRNA.

These are subsequently transported from the nucleus to the cytoplasm, where they are loaded onto ribosomes for protein synthesis or are transported to other compartments, such as P-bodies, to be sequestered and/or degraded [35,36,37]. Although these key mechanisms are conserved, each step in these posttranscriptional processes can be modulated by developmental factors, cellular signaling, or environmental conditions, and in a manner specific to each cell type and tissue by a number of proteins that are able to bind RNA [38,39,40].

To date, it is known that the total number of protein-producing genes in the mammalian genome is about 20,000, but through both pre- and post-transcriptional modifications, we have a complexity many orders of magnitude greater that leads to different and specific cellular functions and dynamic responses to specific external conditions. It is therefore not difficult to verify that the highly regulated processes of mRNA decay and maturation (i.e., mRNA metabolism) should be of critical importance in a wide range of physiopathological contexts.

It is worth mentioning that the transcriptional regulation affects the level of expression of enzymes and proteins involved in different pathways. Transcriptional control requires the transition of specific signals to the cell nucleus, where accurate sets of genes are targeted. In particular, a study published in 2011 demonstrates that under the transcriptional control of MMP-7 (multiple matrix metalloproteinases-7) and TACE (tumor necrosis factor-α convertase), MMP-2 mediates angiotensin II-induced hypertension, suggesting an important regulation point at this level as well [41].

## 5. mRNA Metabolism

RNA metabolism refers to any events in the life cycle of RNA molecules (including their synthesis, folding-unfolding, modification, processing, and degradation).

At the level of all cells in the human body, the proteome, i.e., the complex of proteins expressed by a cell, largely defines its unique functions. Different proteomic profiles and their maintenance characterize the life of most cells. This perfect equilibrium, however, can be disturbed by acute stress phenomena or by long-term or chronic exposure to insults, which often cause irreversible changes in the proteome leading to a dysfunctional phenotype in affected cells and tissues. For example, it is now well recognized that mature cardiomyocytes exhibit a relatively low rate of protein synthesis; conversely, this rate significantly increases during the development of cardiac hypertrophy [42,43].

To date, more than 170 modifications are known to occur at the RNA level in eukaryotes and characterize the topological and chemical properties of these ribose nucleotides, thereby performing their biological functions through post-transcriptional regulation [44]. Modifications at the RNA level were initially studied exclusively in rRNAs, tRNAs, and small nuclear RNAs.

However, with the advent of new sequencing systems, attention has also been turned to mRNAs [45], small nuclear RNA (snRNA) [46], small nucleolar RNA (snoRNA) [46], microRNA (miRNA) [47], long noncoding RNA (lncRNA) [48], and circular RNA (cirRNA) [49]. These different types of RNAs have specific chemical properties, including secondary structure, base pairing, and protein interaction capabilities. Thus, it is not difficult to appreciate that the highly regulated processes of mRNA maturation and decay should have critical importance in a broad array of physiological and disease settings.

**Figure 2:**
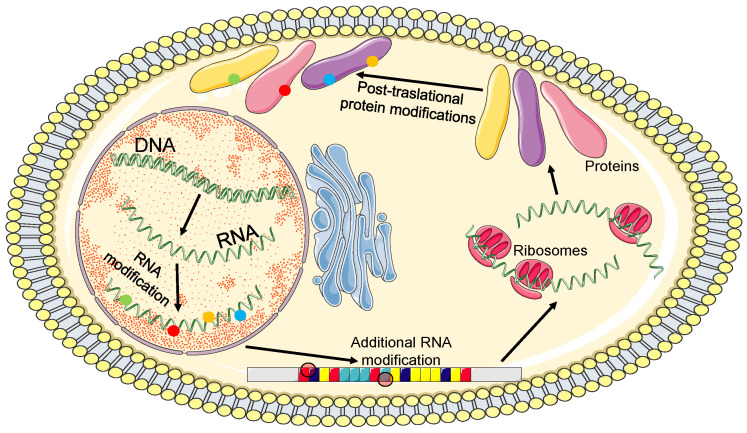
Potential sites of RNA modifications. DNA: deoxyribonucleic acid, RNA: ribonucleic acid. See text for details.

With regard to cardiovascular diseases, a wide range of specific RNA modifications have been identified, including N6-adenosine methylation (m6A), 5-methylcytidine (m5C), 2′-O-ribose-methylation (Nm), pseudouridine (Ψ), N7-methylguanosine (m7G), and N1-adenosine methylation (m1 A) at the expense of different tRNAs, rRNAs, mRNAs, and other noncoding RNAs [50]. These modifications (Figure 2) may function as a novel mechanism in metabolic syndrome (MS), heart failure (HF), coronary heart disease (CAD), and hypertension (Table 1); in particular, they are involved in gene expression by controlling RNA processing, translation, localization, and decay [51].

N6-methyladenosine (m6 A) is a pervasive RNA modification in eukaryotes. It is a hot research topic because of its pivotal role not only in the regulation of gene expression and in the pathogenesis of various diseases, but also in mRNA stability and homeostasis [52].

A high-throughput sequencing analysis of m6A documented that the global level of m6A was reduced in the pericytes of spontaneous hypertensive rats, suggesting a possible role of m6A in hypertension in mammals. In detail, this study revealed the m6A landscape, identifying epi-transcriptomic mechanisms during the development of hypertension. Importantly, several methylation alterations of m6A showed that some genes and pathways co-involved in hypertension were identified by Gene ontology (GO) and Kyoto Encyclopedia of Genes and Genomes (KEGG) analyses [53].

Two different studies have shown how m6 A-SNP (single nucleotide polymorphism) loci are associated with hypertension and have revealed the potential role of m6A in the development of hypertension [54,55]. A cross-sectional analysis (enrolling hypertensive men and women, aged 18 to 80 years) by Marcadenti and coworkers specifically investigated the association of FTO gene rs9939609 and MC4R gene rs17782313 with anthropometric indexes, BP levels, and type 2 diabetes mellitus [54]. They found that common genetic variants of FTO rs9939609 had positive associations with body mass index and neck circumference and MC4R rs17782313 in women, but a negative association with diastolic and mean BP in men with hypertension [54]. The negative association of the common variant of MC4R with BP in men is a novelty, as in previous studies, it had only been described in obese individuals with rare mutations in the MC4R gene and in animal models. However, it is important to emphasize that in this study, the associations of certain anthropometric characteristics with polymorphisms are exploratory for both women and men, as larger samples are needed to confirm the results obtained [54].

Mo and coworkers found different m6A-SNPs that were associated with BP. More specifically, rs9847953 and rs197922 might be functional variants that have the potential to affect the expression of genes (ZNF589 and GOSR2) and BP [55].

As stated by the authors themselves, this study has several limitations. Firstly, the sample sizes were small to identify a large number of associations between m6 A-SNPs and BP levels. Secondly, only a fraction of m6A SNPs (1712 out of 313,000) were examined in this study.

To fully recognize the impact of m6A-SNPs on BP regulation and the effects of a large number of m6A-SNPs on BP levels (especially rare variants) larger data need be evaluated [46]. Collectively, these results reinforce the potential role of m6A in BP levels and BP regulation.

In addition, previous studies have documented that m6A plays a key role in the regulation of downstream molecular events, such as nuclear export, translatability, stability, splicing, and miRNA processing [56]. A second example is the m6 A-SNP rs1801253 in ADRB1, which has the ability to alter the binding of ELF1 and MAX proteins to the STAT regulatory motif, which is localized s in CpG islands and DNase I hypersensitive sites [57].

The association between rs1801253 (known as Arg389Gly) and hypertension has been well studied [58,59]; the individuals homozygous for the Arg389 allele of the beta(1)-adrenergic receptor gene show an increased risk for developing arterial hypertension [57]. A 2020 study revealed that FTO mediated by m6 A plays an essential role in vascular resistance and in the regulation of arterial myogenic contraction [60].

Knockout of EC-specific FTO alleviated obesity-induced vascular resistance and increased BP. In detail, Kruger and co-workers demonstrated that loss of endothelial FTO protected from HFD-induced hypertension and increases heart rate. Furthermore, analysis of vascular reactivity in resistance arteries showed that loss of endothelial FTO did not significantly affect depolarization-induced contractility, endothelium-derived hyperpolarization (EDH) signaling, or acetylcholine-induced vasodilation [60].

In human and mouse vessels, endothelial FTO inhibits the expression of lipocalin-like prostaglandin D synthase (L-PGDS) in an m6-dependent manner, resulting in a decrease in PGD2 (prostaglandin decrease in PGD2 (prostaglandin D2) [60].

This mechanism is supported by other previous studies where they confirmed that PGD2 can activate the G-protein-coupled receptor on the vascular smooth muscle membrane and regulate the diastolic movement of VSMCs [61].

On the other hand, VSMCs play a central role in the regulation of vascular resistance and diastolic BP. m6A-mediated changes have a role in the proliferation, migration, and phenotypic transformation of VSMCs [62,63]. VSMCs dysfunction is a common pathophysiological mechanism of cardiovascular diseases, including essential hypertension, pulmonary hypertension, atherosclerosis, and aneurysm disease.

A very recent Italian study published in August 2022 identified five miRNAs, which are involved in the onset of salt-sensitive hypertension, including miR-23a that was bioinformatically predicted to target sodium-hydrogen exchanger 1 (NHE1) mRNA [64]. Specifically, Lombari and colleagues identified miRNA expression profiles in isolated medullary thick ascending limbs (mTALs) from high sodium intake-induced hypertensive rats versus their normotensive counterparts. In vitro validation was carried out in rat mTAL cells. Results demonstrated that miRNA-23a is downregulated in the mTAL of high sodium intake-induced hypertensive rats whereas NHE1 is upregulated. Consistently, transfection of a miRNA-23a mimic in an mTAL cell line, using a viral vector, resulted in NHE1 downregulation. In other words, the downregulation of miRNA-23a in humans with hypertension support the hypothesis of a potential role of miRNA-23a in the regulation of mTAL function following high salt intake [64].

Others studies investigated the relationship between aryl hydrocarbon receptor nuclear translocators such as Arntl, also known as BMAL1, rs6486121, and clock circadian regulator (Clock) T3111C with hypertension [65,66,67]. In a case-control study, 172 subjects were recruited, of which 86 were hypertensive and 86 were non-hypertensive controls. Arntl mRNA expression levels in peripheral blood mononuclear cells were determined by quantitative real-time polymerase chain reaction (PCR). The mRNA expression of Arntl was downregulated in the hypertensive cases when compared with controls in women suggesting that Arntl mRNA expression plays a possible role in the progression of hypertension [68].

Chunlan Liu and colleagues explored the associations of Filamin A (FLNA) and Filamin B (FLNB) variants with hypertension. Filamin A and filamin B were involved in cell migration [69,70] and vascular development and remodeling [70]. They analyzed the associations of two SNPs at the FLNA level and five SNPs in the FLNB gene with hypertension. They performed two case-control studies for a total of 2012 and 2210 hypertensive and normotensive cases. FLNA rs2070816 (CT + TT vs. CC) and rs2070829 (CG + GG vs. CC) were significantly associated with the hypertensive status in young subjects (<55 years group) and FLNB rs839240 (AG + GG vs. AA) was significantly associated with hypertension in females. Moreover, rs2070829 GG genotype carriers showed a higher risk of hypertension than CC/CG in males [71].

These findings clearly support the genetic contribution of FLNA and FLNB to hypertension, with differentially mRNA expression.

The deamination of adenosine in inosine is one of the most widespread epitranscriptomic modifications known to date. This modification can recode mRNAs to translate new protein variants. To date, ADAR2 is believed to act mainly in the brain and its clinical significance has so far been linked to functions related to the nervous system. However, a study published in 2018 shows that ADAR 2 activity in vascular tissues is 10-fold higher when editing in sites such as FLNA, which regulate vascular constriction and protect against cardiac remodeling. In particular, when throwing in vivo models, results showed that the absence of FLNA editing causes inhibition of normal aortic relaxation, hypercontraction of smooth muscle cells, and increased DBP, demonstrating that decreased FLNA editing can also contribute to cardiovascular diseases [72].

In summary, this study conducted on FLNA demonstrated a causal relationship between editing of the mRNA that encodes actin crosslinking protein Filamin A, which mediates a Q-to-R transition in the interactive C-terminal region, and the development of cardiovascular disease, indicating that a single epitranscriptomic RNA modification can maintain cardiovascular health [72].

**Table 1:** Features of main studies evaluating the link between mRNA metabolism and hypertension. Arntl: aryl hydrocarbon receptor nuclear translocator such as FLNA: filamin A, FLNB: filamin B, FTO: fat mass and obesity associated gene, HSD rats: high sodium intake induced hypertensive rats, MC4R: Melanocortin 4 Receptor gene, m6 A-SNP: N6-methyladenosine single nucleotide polymorphism, miR-23a: microRNA-23a, mRNA: messenger ribonucleic acid, mTAL: medullary thick ascending limb, NHE1: sodium hydrogen exchanger 1; nucleotides = A: adenine, C: cytosine, G: guanine, T: thymine.

Author	Modification	Function
Marcadenti, A et al. [54]	FTO gene rs9939609 and MC4R gene rs17782313	m6 A-SNP loci are associated with hypertension
Bengtsson, K. et al. [57]	Arg389 variant of the beta(1)-adrenergic receptor gene	Homozygous for the Arg389 allele of the beta(1)-adrenergic receptor gene is at increased risk to develop hypertension
Lombari, P. et al. [64]	miR-23a	Expression is downregulated in the mTAL of HSD rats whereas NHE1 is upregulated
Fang, Z. et al. [68]	Arntl rs6486121	mRNA expression of Arntl was downregulated in hypertension cases compared with controls in women
Liu, C. et al. [71]	FLNA rs2070816 (CT + TT vs. CC) and rs2070829 (CG + GG vs. CC)	They were significantly associated with hypertension in <55 years group
	FLNB rs839240 (AG + GG vs. AA)	It was significantly associated with hypertension in females

## 6. Clinical Trials

Two ongoing clinical trials are specifically evaluating a subcutaneous RNAi therapeutic targeting liver-expressed angiotensinogen for the treatment of hypertension.

The phase 2 KARDIA-1 study is a randomized, double-blind (DB), placebo-controlled, dose-ranging trial to evaluate the efficacy and safety of ALN-AGT01 as a monotherapy in adults with mild to moderate hypertension. The trial will evaluate ALN-AGT01 as a monotherapy in different doses administered quarterly and semiannually. The purpose of this clinical trial is to evaluate the effect of ALN-AGT01 on SBP and DBP and to characterize the safety and pharmacodynamics (PD) effects of ALN-AGT01. The primary outcome is the change in 24 h SBP from baseline at month 3 determined using 24 h ambulatory BP monitoring (ABPM). Secondary outcome measures include the changes from baseline at months 3 and 6 in office SBP, the changes from baseline at month 6 in 24 h mean SBP, the proportion of patients with 24 h mean SBP assessed by ABPM < 130 mmHg and/or reduction ≥ 20 mmHg without additional antihypertensive medications at Month 6, the time-adjusted changes from baseline in 24 h mean SBP and DBP, change from baseline in 24-h mean diastolic BP, the changes from baseline in office SBP and DBP, the changes in serum angiotensinogen, and the changes from baseline in daytime and nighttime SBP and diastolic BP by ABPM (including Dipping Pattern). A daytime mean SBP ≥ 135 mmHg and ≤160 mmHg by ABPM in the absence of antihypertensive drugs is the only inclusion criterion. Exclusion Criteria include orthostatic hypotension, secondary hypertension, the estimated glomerular filtration rate (eGFR) of ≤30 mL/min/1.73 m^2^, alanine aminotransferase (ALT) or aspartate aminotransferase (AST) > 2 × upper limit of normal, elevated potassium > 5 mEq/L, received an investigational agent within the last 30 days, type 1 diabetes or poorly controlled type 2 diabetes mellitus, newly diagnosed type 2 diabetes mellitus, history of any cardiovascular event within 6 months prior to randomization, history of intolerance to subcutaneous injection. The study is currently ongoing and will be completed in 2024 (https://clinicaltrials.gov/ct2/show/NCT04936035 [Accessed on 1 December 2022]).

The phase 2 KARDIA-2 study is a randomized, double-blind (DB), placebo-controlled. The main aim of this trial is to evaluate the effect of zilebesiran on SBP and DBP and to evaluate the safety and PD effects of this novel treatment as add-on therapy in patients with not adequately controlled hypertension. The primary outcome is the change from baseline at month 3 in 24 h mean SBP assessed by ABPM. Secondary outcome measures include the change from baseline at month 3 in office SBP and DBP, time-adjusted change from baseline through month 6 in BP, 24 h mean SBP (as assessed by ABPM), the proportion of patients with 24 h mean SBP assessed by ABPM < 130 mmHg and/or reduction from baseline ≥ 20 mmHg without escape antihypertensive medication at month 6, change in 24 h mean SBP and DBP, change in office and ambulatory SBP and DBP, and change from baseline in serum angiotensinogen (AGT). The inclusion criteria are adult male or female subjects (18–75 years old) with office SBP at screening ≥ 155 mmHg and ≤180 mmHg for patients with untreated hypertension (or ≥145 mmHg and ≤180 mmHg for patients treated with BP lowering drugs), and with 24 h uncontrolled SBP (SBP > 130 mmHg and ≤160 mmHg by ABPM after at least 4 weeks of run-in). The exclusion criteria are secondary hypertension, orthostatic hypotension, eGFR of <30 mL/min/1.73 m^2^, elevated potassium >5 mEq/L, newly diagnosed type 2 diabetes mellitus, type 1 diabetes mellitus or poorly controlled, type 2 diabetes mellitus, and history of any cardiovascular event within 6 months prior to randomization or history of intolerance to subcutaneous injection. The study started in 2021 and will be completed in July 2024 (https://clinicaltrials.gov/ct2/show/NCT05103332 [Accessed on 1 December 2022]).

## 7. Conclusions

In the last few years, alterations of the mRNA metabolism have been evaluated as contributors to the pathogenesis of hypertension. As aforementioned, several RNA modifications have been identified, including m6A, m5C, Nm, Ψ, m7G, and m1 A [50,51].

These mechanisms support the notion that a pharmacological modulation of RNA metabolism might be useful to reduce BP among hypertensive patients. In this context, it is worth noting that RNAi drugs act through a natural cellular process of gene silencing that aims to “silence” mRNA, preventing gene expression and thus blocking the production of the target protein. In essence, the siRNA molecules in the drug act upstream, deactivating the genetic precursors that code for disease-causing proteins, thereby preventing their progression even before they begin.

In this context, ALN-AGT01 is an investigational RNAi subcutaneously administered angiotensinogen-targeted (AGT) drug under development for the treatment of hypertension in low pharmaceutical adherence populations. As aforementioned, two clinical phase 2 trials are currently evaluating safety and efficacy of ALN-AGT01. To date, an interim analysis from a phase 1 study of ALN-AGT01 demonstrated a sustained effect on angiotensinogen (circulating angiotensinogen reduced by >90% for 6 months after a single subcutaneous dose of 800 mg) [73]. A sustained reduction of BP was also observed, using ABPM [73]. Moreover, ALN-AGT01 was well tolerated; only mild-to-moderate injection site reactions (*n* = 5/56) were recorded and no serious adverse events were observed [73].

In conclusion, RNA-based medicine with the development of novel therapeutic strategies has the potential to offer advantages over existing treatments, including the improvement in treatment adherence through providing a simplified therapeutic regimen. However, efficacy and safety of these novel agents remains to be determined among specific subpopulations of hypertensive patients, such as those with diabetes, previous vascular events, and chronic kidney disease. Finally, the prognostic impact of these therapeutic regimens remains unanswered.

## Data Availability

Not applicable.
